# Constipation‐associated factors in outpatients with schizophrenia: A multicenter questionnaire survey

**DOI:** 10.1002/npr2.12464

**Published:** 2024-07-03

**Authors:** Taro Tazaki, Hiroki Yamada, Ryotaro Sato, Hiroki Ishii, Shutaro Sugita, Haruka Yanagihara, Dan Nakamura, Osamu Takashio, Atsuko Inamoto, Akira Iwanami

**Affiliations:** ^1^ Department of Psychiatry, Graduate School of Medicine Showa University Tokyo Japan; ^2^ Department of Psychiatry, School of Medicine Showa University Tokyo Japan; ^3^ Showa University Karasuyama Hospital Tokyo Japan; ^4^ Department of Psychiatry Showa University Northern Yokohama Hospital Tokyo Japan; ^5^ Shinrin Koen Mental Clinic Tokyo Japan; ^6^ Department of Psychiatry Showa University East Hospital Tokyo Japan

**Keywords:** antipsychotic agents, constipation, outpatients, psychotropic drugs, schizophrenia

## Abstract

Constipation is a prevalent gastrointestinal disorder that affects people globally, decreasing their quality of life and life expectancy. Individuals with schizophrenia often suffer from constipation, which could be a result of the illness itself or the side effects of psychotropic medications. However, little research has been conducted on factors contributing to constipation in individuals with schizophrenia. To address this issue, we conducted a survey using self‐administered questionnaires and medical records to identify factors associated with constipation in psychiatric outpatients. This study included 399 patients with schizophrenia, resulting in a high prevalence of constipation (43.4%). The analysis suggested that female gender, the doses of antiparkinsonian medications, and benzodiazepine sleeping pills may be associated with constipation.

## Abstract 2

Individuals with schizophrenia often suffer from constipation, which could be a result of the illness itself or the side effects of psychotropic medications. We conducted a survey using self‐administered questionnaires and medical records to identify factors associated with constipation in psychiatric outpatients. The analysis suggested that female gender, the doses of antiparkinsonian medications, and benzodiazepine sleeping pills may be associated with constipation.

## Graphical abstract

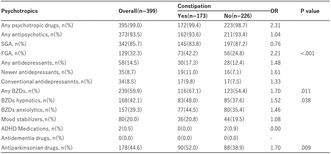

## 1 INTRODUCTION

Constipation is a common functional gastrointestinal disorder that affects a considerable number of people globally. According to the National Nutrition and Lifestyle Survey conducted by the Ministry of Health, Labour, and Welfare, constipation occurs in 2.5% of males and 4.6% of females in Japan.^1^ In the United States, approximately 14% of the population has chronic constipation, with an increased incidence among women, older adults, and individuals of lower socioeconomic status.^2^ A large population‐basedcohort study by Oh et al. for those experiencing constipation revealed that 24.0% of participants (1128 of 4702) had chronic idiopathic constipation, whereas 47.8% (2246 of 4702) used medications to alleviate constipation.^3^ The study also reported that individuals with psychiatric disorders are likely to require medical attention for constipation (odds ratio [OR]: 1.25, 95% confidence interval [CI]: 1.09–1.44).^3^

It is essential to understand that constipation is not only a common condition but can also be life‐threatening. Chang et al. investigated the impact of various gastrointestinal dysfunctions on survival.^4^ The findings indicated that individuals with chronic constipation are at risk for decreased survival.^4^ Another study by Sumida et al. found that constipation or laxative use was independently associated with an increased risk of chronic kidney disease, ischemic stroke, and all‐cause mortality.^5^ Furthermore, constipation has been reported to significantly reduce the quality of life to an extent comparable to that of other physical and mental illnesses.^6^

Constipation is a common symptom in people with schizophrenia, with a reported period prevalence of 36.3% over 2 years.^7^ The study conducted by Jessurun et al. found that constipation was more prevalent in patients with schizophrenia and other psychiatric disorders than in the general population.^8^ In their study, 20.3% of 4728 patients had constipation.^8^ Among patients aged ≥60 years, schizophrenia was significantly associated with an increased likelihood of constipation (OR: 5.72, 95% CI: 2.01–16.28).^8^ Individuals with schizophrenia have a life expectancy that is 15 years shorter than that of the general population.^9^ Constipation may be one of the causes of short life expectancy in patients with schizophrenia.

Schizophrenia can be treated using various psychotropic drugs. According to Xu et al., constipation is a common side effect of antipsychotic medications, affecting >50% of patients who use them.^10^ These drugs block dopamine receptors and exert anticholinergic and antihistaminic effects. In some cases, multiple categories of psychotropic medication are prescribed. Antiparkinsonian drugs with anticholinergic effects are also used to prevent or treat extrapyramidal symptoms. The anticholinergic side effects of antipsychotics include dry mouth due to decreased salivation. Reduced intestinal mobility can cause constipation, while inhibited visual accommodation, increased pupil size, and tachycardia can lead to blurred vision.^11^ First‐generation H1‐antihistamines cause drowsiness, fatigue, headache, memory problems, and other side effects like sedation, increased appetite, dry mouth, dry eyes, visual disturbances, constipation, urinary retention, and erectile dysfunction.^12^ Naitou et al. also demonstrated that dopamine in the lumbosacral defecation center causes strong propulsive colon movements via dopamine D2‐like receptors in rats.^13^ The study supported the theory that the descending pathway responsible for pain suppression also regulates the defecation reflex.^13^ Psychotropic drugs have various pharmacological effects on constipation, and their possible influence on constipation in patients with schizophrenia must be considered.

Constipation is a frequently occurring problem in patients with schizophrenia. However, few guidelines related to schizophrenia have mentioned this.^14^ There is inadequate evidence regarding risk factors for constipation in patients with schizophrenia. To address this issue, we conducted a study that examined the demographic and clinical factors associated with constipation in outpatients with schizophrenia, using a self‐administered questionnaire at four different facilities.

## 2 METHODS

### 2.1 Participants

In this multicenter questionnaire survey‐based study, the participants were outpatients who visited the Showa University Karasuyama Hospital (a psychiatric hospital), Showa University Northern Yokohama Hospital (a general hospital with psychiatric beds), and Shinrin‐Koen Mental Clinic (a psychiatric clinic) between January and April 2020. From January to April 2023, the same survey was conducted at the Showa University East Hospital, a general hospital without psychiatric beds. We included those who received one or more diagnoses according to the International Classification of Diseases 10th Revision F0 to F9 (F0: organic mental disorders, including symptomatic; F1: mental and behavioral disorders due to psychoactive substance use; F2: schizophrenia, schizotypal disorder, and delusional disorder; F3: mood [affective] disorder; F4: neurotic disorder, stress‐related disorder, and somatoform disorder; F5: physiological disorders and behavioral syndromes related to physical factors; F6: personality and behavioral disorders in adults; F7: intellectual disability; F8: disorders of psychological development; and F9: behavioral and emotional disorders that usually occur in childhood and adolescence) and G4 (interictal and seizure disorders). A principal diagnosis was established for comorbid cases. The exclusion criteria were those deemed by the attending physician to lack decision‐making capacity and those who did not consent to participate in the study.

### 2.2 Questionnaire and definition of constipation

Tables 1 and 2 present the self‐administered questionnaires used in this study. The questionnaire collected information regarding patient demographics, lifestyle, and bowel movements. Constipation was determined according to the Rome IV criteria, the diagnostic criteria for chronic and functional constipation developed by the Rome Committee.^15^ The questionnaire was designed so that, depending on how the participants answered it, we could determine whether they met the Rome IV criteria. This study defined constipation as meeting the requirements of Rome IV, using laxatives, or both.

**TABLE 1:** Questionnaires distributed (first half).

Age	Years old
Sex	M/F
Height	Cm
Body weight	kg
How many years has it been since you first became ill?	years
How many years has it been since you first started treatment?	years
Do you live alone?	Yes/No
How many days per week do you exercise?	days
How many days per week do you eat out?	days
Are you currently feeling stressed?	Yes/No
Do you drink alcohol?	Yes/No
Do you smoke?	Yes/No

**TABLE 2:** Questionnaires distributed (second half).

Straining (during at least 25% of bowel movements)	Yes/No
Hard stools at least 25% of the time	Yes/No
A feeling of incomplete evacuation (at least 25% of the time)	Yes/No
Sensation of blockage or obstruction in the anus or rectum area (at least 25% of the time)	Yes/No
Manual efforts to enable the passage of stool at least 25% of the time (e.g., digital evacuation)	Yes/No
Less than three spontaneous bowel movements (SBM)/week	Yes/No
Prescribed laxatives at another hospital	Yes/No
Use of over‐the‐counter laxatives	Yes/No
Frequency of defecation/week	Times
Recent stool condition	Bristol Stool Form Scale

### 2.3 Procedure

The questionnaire was administered only once and was completed on‐site when all participants who consented to the study visited one of the facilities involved. Medical records were used to collect information on the participants' illness, duration of treatment, medication history, and the type and quantity of medications taken on the day the questionnaire was administered.

### 2.4 Dosage considerations

Specific drug categories, such as chlorpromazine equivalent, imipramine equivalent, and biperiden equivalent, were used to estimate the dosage. These metrics help to study the correlation between drug dosage and the likelihood of laxative use. However, not all drugs have such metrics, and no universal index for all medicines can be combined and measured. Additionally, some drug categories do not have indices; therefore, we developed our own “clinical dose ratio (CDR).” The CDR is a simple representation of the dosage of a drug, calculated using the maximum recommended dosage listed in the package insert in Japan. The maximum dosage in Japan was considered to be 1, and the ratio of the actual dosage to this dosage was calculated. Although this method has limited academic value, we found it practical for visualizing and understanding drug dosage in real‐world clinical situations. In this study, we calculated the total dose for each drug category by adding the CDRs. The upper limits of the Japanese package inserts were used to calculate the CDR (e.g., risperidone 6 mg = 6/6 = 1, 3 mg = 3/6 = 0.5; quetiapine [fast‐release tablet] 750 mg = 750/750 = 1, 500 mg = 500/750 = 0.67; escitalopram 10 mg + mirtazapine 15 mg = 10/20 + 15/45 = 0.5 + 0.33 = 0.83). The dosage of quetiapine extended‐release tablets, which are indicated only for bipolar disorder in Japan, was calculated separately from that of quetiapine fast‐release tablets.

### 2.5 Statistical analysis

A descriptive analysis was conducted to summarize the characteristics of the participants. The study participants were divided into two groups: those with and without constipation. A between‐group analysis was performed on various study variables, including age, sex, duration of illness, duration of treatment, body mass index (BMI), International Classification of Diseases 10th Revision diagnosis, awareness of stress, alcohol consumption, smoking, number of eating‐out days per week, number of exercise days per week, and medications. Nominal variables were analyzed using the chi‐square test or Fisher's exact probability test, whereas continuous variables were analyzed using the Mann– Whitney U test. Logistic regression was used to determine the relationship between constipation and factors such as age, sex, duration of illness, duration of treatment, BMI, stress awareness, alcohol consumption, smoking, number of eating‐out days per week, number of exercise days per week, and medications.

To investigate the association between psychotropic medication dosage and constipation, this study analyzed medication types, including first‐generation antipsychotics (FGAs), second‐generation antipsychotics (SGAs), antiparkinsonian drugs, new and conventional antidepressants, benzodiazepine (BZD) anxiolytics, BZD sleeping pills, mood stabilizers, attention‐deficit/hyperactivity disorder medications, and antidementia medications, using CDRs. Second‐generation antipsychotics were defined as risperidone, olanzapine, quetiapine, perospirone, aripiprazole, paliperidone, blonanserin, brexpiprazole, and lurasidone, whereas others were considered FGAs. The new antidepressants used were fluvoxamine, paroxetine, milnacipran, duloxetine, mirtazapine, escitalopram, and vortioxetine, whereas the others were classified as conventional antidepressants. Non‐BZDs with gamma‐aminobutyric acid receptor agonists were included among the BZDs. Mood stabilizers included lithium carbonate, valproate, carbamazepine, lamotrigine, and quetiapine extended‐release tablets. Quetiapine extended‐release tablets were considered mood stabilizers in this study because their pharmacokinetics and maximum dosage differ from those of fast‐release tablets, and they are not indicated for schizophrenia in Japan. None of the participants in this study were administered clozapine. The laxatives used for defecation control in this study were sennoside, senna, senna extract, magnesium oxide, mosapride citrate, pantethine, lubiprostone, elobixivat, linaclotide, and other herbal medicines that have been used for defecation control based on medical records. Because it was difficult to clarify the use of laxatives on an abortive basis, prescriptions of laxatives on regular medications were counted instead. The significance level was set at 0.05. Microsoft Excel 2019 (Microsoft Corp., Redmond, WA) and SPSS (version 25.0; IBM Corp., Armonk, NY) were used for all statistical analyses.

## 3 RESULTS

### 3.1 Demographic and clinical characteristics of the participants

In total, 2440 participants were recruited, and 1856 individuals (mean age, 48.2 years; 650 men) were eligible and included in the study. Of all the patients, 399 individuals diagnosed with schizophrenia were chosen for the analysis.

Table 3 presents the demographic and clinical characteristics of the participants. The sex proportion was 44.6% (178) males and 55.4% (221) females, with a slight female predominance. The average age of the participants was 49.3 (standard deviation [SD], 12.5) years, with intermediate disease and treatment durations of 20.9 (SD, 11.6) and 19.5 (SD, 11.3) years, respectively. The average height, weight, and BMI were 163.1 (SD, 8.4) cm, 67.8 (SD, 15.6) kg, and 25.4 (SD, 4.9) kg/m^2^, respectively. Of the 399 patients with schizophrenia, 173 had constipation, and 226 did not, resulting in a constipation (+) rate of 43.4% (173/399).

**TABLE 3:** Demographic and clinical characteristics of participants.

Characteristics	Overall (*n* = 399)	Constipation		OR	*p* Value
		(+) (*n* = 173)	(−) (*n* = 226)		
Mean age (SD), years	49.3 (17.0)	50.3 (13.4)	48.5 (11.7)		
Male, *n* (%)	178 (44.6)	63 (36.4)	115 (50.9)	0.55	0.003*
Mean duration of illness (SD), y^a^	20.9 (11.6)	21.6 (12.4)	20.3 (11.8)		
Mean duration of treatment (SD), y^b^	19.5 (11.3)	20.0 (11.9)	19.2 (11.6)		
Mean stature (SD), cm^c^	163.1 (8.4)	162.2 (8.3)	163.8 (8.4)		0.005*
Mean body weight (SD), kg^d^	67.8 (15.6)	65.6 (14.2)	69.5 (10.6)		
Mean BMI (SD)^e^	25.4 (4.9)	24.8 (4.5)	25.8 (5.2)		

*Note*: Data are presented as numbers (percentage) and mean (SD). We performed the chi‐square test for nominal variables and Mann–Whitney's *U* test for continuous variables. Abbreviations: BMI, body mass index; OR, odds ratio; SD, standard deviation. ^a–e^Participants without missing values (^a^ *n* = 385, ^b^ *n* = 386, ^c^ *n* = 393, ^d^ *n* = 384, ^e^ *n* = 384).

* *p* < 0.01.

### 3.2 Between‐group comparison of backgrounds

Table 3 compares the constipation (+) and the constipation (−) groups regarding various background characteristics. The constipation (+) group had a sex ratio of 36.4% males and 63.6% females, whereas the constipation (−) group had a sex ratio of 50.9% males and 49.1% females. There were significantly more women in the constipation (+) group (*p* = 0.003) than in the constipation (−) group. The constipation (+) group had a mean height of 162.2 (SD, 8.3) cm, whereas the constipation (−) group had a mean height of 163.8 (SD, 8.4) cm, indicating a significant difference in height between the two groups (*p* = 0.005). There were no significant differences in age, disease duration, treatment duration, weight, or BMI.

### 3.3 Between‐group comparison of lifestyle

Table 4 compares the lifestyle variables between the constipation (+) and the constipation (−) groups. Perceived stress was significantly higher in the constipation (+) group (64.2%) than in the constipation (−) group (48.7%) (*p* = 0.002). There was a significant difference in drinking habits between the constipation (+) group (12.7%) and the constipation (−) group (21.7%); the constipation (+) group had lower rates of drinking (*p* = 0.020) than the constipation (−) group. The constipation (+) group defecated significantly less frequently (8.1 times per week) than the constipation (−) group (9.4 times per week) (*p* < 0.001). The percentage of patients reporting subjective symptoms during defecation was significantly higher in the constipation (+) group (69.9%) than in the constipation (−) group (23.5%) (*p* < 0.001). There were no noticeable differences in lifestyle habits between the two groups.

**TABLE 4:** Lifestyle factors between constipation (+) and constipation (−) groups.

Characteristics	Overall (*n* = 399)	Constipation		OR	*p* Value
		(+) (*n* = 173)	(−) (*n* = 226)		
Living alone, *n* (%)	101 (25.3)	41 (23.7)	61 (27.3)	0.84	
Habit of eating out, *n* (%)	229 (57.4)	96 (55.5)	133 (58.8)	0.87	
Mean frequency of eating out/week (SD)	1.2 (1.7)	1.2 (1.6)	1.3 (1.7)		
Habit of exercise, *n* (%)	211 (52.9)	83 (48.0)	128 (56.6)	0.70	
Mean frequency of exercise/week (SD)	1.7 (1.7)	1.5 (2.1)	1.9 (2.3)		
Feeling stressed, *n* (%)	221 (55.4)	111 (64.2)	110 (48.7)	1.88	0.002**
Alcohol intake, *n* (%)	79 (17.8)	22 (12.7)	49 (21.7)	0.52	0.020*
Smoking, *n* (%)	85 (21.3)	39 (22.5)	46 (20.4)	1.13	
Frequency of defecation/week (SD)	8.5 (6.4)	8.1 (8.0)	9.4 (6.4)		<0.001***
Subjective symptoms during defecation, *n* (%)	174 (43.6)	121 (69.9)	53 (23.5)	7.60	<0.001***

*Note*: Data are presented as numbers (percentage) and mean (SD). We performed the chi‐square test for nominal variables and Mann–Whitney's *U* test for continuous variables. Abbreviations: OR, odds ratio; SD, standard deviation. *p < 0.05, **p < 0.01, ***p < 0.001.

### 3.4 Between‐group comparison of prescription rates

Table 5 compares the prescription rates of psychotropic drugs prescribed for the constipation (+) and the constipation (−) groups. The prescription rate for FGA was 42.2% in the constipation(+) group and 24.8% in the constipation(−) group, with the constipation group being significantly more common (*p* < 0.001). The prescription rate for any BZDs was 67.7% in the constipation(+) group and 54.4% in the constipation(−) group, with the constipation group being significantly more common (*p* = 0.011). The prescription rate for BZD hypnotics was 48.0% in the constipation (+) group and 37.5% in the constipation (−) group, with the constipation group being significantly more common (*p* = 0.038). The prescription rate for Antiparkinsonian drugs was 52.0% in the constipation(+) group and 38.9% in the constipation(−) group, with the constipation group being significantly more common (*p* = 0.009).

**TABLE 5:** Psychotropics prescription rates between the constipation(+) and constipation(−) groups.

Psychotropics	Overall (*n* = 399)	Constipation		OR	*p* Value
		(+) (*n* = 173)	(−) (*n* = 226)		
Any psychotropic drugs, *n* (%)	395 (99.0)	172 (99.4)	223 (98.7)	2.31	
Any antipsychotics, *n* (%)	373 (93.5)	162 (93.6)	211 (93.4)	1.04	
SGA, *n* (%)	342 (85.7)	145 (83.8)	197 (87.2)	0.76	
FGA, *n* (%)	129 (32.3)	73 (42.2)	56 (24.8)	2.21	<0.001
Any antidepressants, *n* (%)	58 (14.5)	30 (17.3)	28 (12.4)	1.48	
Newer antidepressants, *n* (%)	35 (8.7)	19 (11.0)	16 (7.1)	1.61	
Conventional antidepressannts, *n* (%)	34 (8.5)	17 (9.8)	17 (7.5)	1.33	
Any BZDs, *n* (%)	239 (59.9)	116 (67.1)	123 (54.4)	1.70	0.011
BZDs hypnotics, *n* (%)	168 (42.1)	83 (48.0)	85 (37.6)	1.52	0.038
BZDs anxiolytics, *n* (%)	157 (39.3)	77 (44.5)	80 (35.4)	1.46	
Mood stabilizers, *n* (%)	80 (20.0)	36 (20.8)	44 (19.5)	1.08	
ADHD medications, *n* (%)	2 (0.5)	0 (0.0)	2 (0.9)	0.00	
Antidementia drugs, *n* (%)	0 (0.0)	0 (0.0)	0 (0.0)	‐	
Antiparkinsonian drugs, *n* (%)	178 (44.6)	90 (52.0)	88 (38.9)	1.70	0.009

*Note*: Data are presented as numbers (percentages). For nominal variables, we used the chi‐squared test.

Abbreviations: ADHD, attention‐deficit/hyperactivity disorder; BZD, benzodiazepine; FGA, first‐generation antipsychotics; OR, odds ratio; SGA, second‐generation antipsychotics.

### 3.5 Between‐group comparison of CDRs

Table 6 compares the CDRs of psychotropic drugs prescribed for the constipation (+) and constipation (−) groups. The constipation (+) group had significantly higher total CDRs for psychotropic medications, including antiparkinsonian drugs, than the constipation (−) group (2.8 [SD, 2.0] vs. at 2.0 [SD, 1.6]; *p* = 0.011). The constipation (+) group had a significantly higher total CDR for all antipsychotics than the constipation (−) group (1.2 [SD, 1.0] vs. 0.9 [SD, 0.8]; *p* = 0.003). The constipation (+) group had a significantly higher CDR for FGA than the constipation (−) group (0.3 [SD, 0.6] vs. 0.1 [SD, 0.4]; *p* = 0.009). There was no significant difference in SGA between the two groups. The CDR for antiparkinsonian drugs was significantly higher in the constipation (+) group than in the constipation (−) group (0.24 [SD, 0.32] vs. 0.17 [SD, 0.28]; *p* = 0.016). The constipation (+) group had a significantly higher CDR for BZDs, which is the sum of anxiolytics and sleeping pills, than the constipation (−) group (1.1 [SD, 0.8] vs. 0.7 [SD, 0.9]; *p* = 0.002). The CDR for BZD sleeping pills was 0.8 (SD, 1.0) for the constipation (+) group, whereas it was 0.5 (SD, 0.7) for the constipation (−) group. This indicated that the CDR for BZD sleeping pills was significantly higher in the constipation (+) group than in the constipation (−) group (*p* = 0.001). However, there was no significant difference in the CDR for BZD anxiolytics between the two groups. The CDRs for both antidepressants and mood stabilizers were similar between the two groups. However, no significant differences were observed between groups.

**TABLE 6:** Between‐group comparison on CDRs.

Psychotropics	Overall (*n* = 399)	Constipation		*p* Value
		(+) (*n* = 173)	(−) (*n* = 226)	
Any psychotropic drugs' CDR (SD)	2.3 (1.8)	2.8 (2.0)	2.0 (1.6)	0.011*
Any antipsychotics' CDR (SD)	1.1 (0.9)	1.2 (1.0)	0.9 (0.8)	0.003**
SGAs' CDR (SD)	0.8 (0.7)	0.9 (0.8)	0.8 (0.6)	
FGAs' CDR (SD)	0.2 (0.5)	0.3 (0.6)	0.1 (0.4)	0.009**
Antiparkinsonian drugs' CDR (SD)	0.2 (0.3)	0.24 (0.32)	0.17 (0.28)	0.016*
Any antidepressants' CDR (SD)	0.1 (0.3)	0.1 (0.4)	0.1 (0.3)	
Newer antidepressants' CDR (SD)	0.1 (0.2)	0.1 (0.2)	0.1 (0.2)	
Conventional antidepressants' CDR (SD)	0.0 (0.2)	0.0 (0.2)	0.0 (0.1)	
Any BZDs' CDR (SD)	0.9 (1.0)	1.1 (0.8)	0.7 (0.9)	0.002**
BZDs sleeping pills' CDR (SD)	0.6 (0.8)	0.8 (1.0)	0.5 (0.7)	0.001**
BZDs anxiolytics' CDR (SD)	0.2 (0.4)	0.2 (0.4)	0.2 (0.5)	
Mood stabilizers' CDR (SD)	0.1 (0.3)	0.1 (0.3)	0.1 (0.2)	
ADHD medications' CDR (SD)	0	0	0	
Antidementia drugs' CDR (SD)	0	0	0	

*Note*: Data are presented as mean (SD). We performed the Mann–Whitney's *U* test for continuous variables. Abbreviations: ADHD, attention‐deficit/hyperactivity disorder; BMI, body mass index; BZD, benzodiazepine; FGA, first‐generation antipsychotics; OR, odds ratio; SD, standard deviation; SGA, second‐generation antipsychotics. *p < 0.05, **p < 0.01.

### 3.6 Factors associated with constipation

According to the logistic regression analysis presented in Table 7, female sex (OR: 2.084, 95% CI: 1.352–3.211, *p* = 0.001), CDR for antiparkinsonian drugs (OR: 2.364, 95% CI: 1.133–4.936, *p* = 0.022), and CDR for BZD sleeping pills (OR: 1.525, 95% CI: 1.174–1.982, *p* = 0.002) were significantly associated with constipation.

**TABLE 7:** Factors associated with constipation.

Characteristics	B (SE)	Wald	OR	95% CI	*p* value
Gender (female)^a^	0.734 (0.221)	11.064	2.084	1.352–3.211	0.001**
Antiparkinsonian drugs' CDR^b^	0.860 (0.375)	5.260	2.364	1.133–4.930	0.022*
BZD sleeping pills' CDR^b^	0.422 (0.134)	9.990	1.525	1.174–1.982	0.002**

*Note*: Multiple logistic regression analysis forward stepwise entry method. Abbreviations: BZD, benzodiazepine; CDR, clinical dose ratio; CI, confidence interval; OR, odds ratio; SE, standard error.

^a^ Male coded 0, female coded 1.

^b^ Value per one CDR increase.

*p < 0.05, **p < 0.01.

## 4 DISCUSSION

In this study, we examined the demographic and clinical factors associated with constipation in outpatients with schizophrenia using a self‐administered questionnaire and clinical records at four different facilities. We found that 43.4% (173/399) of the patients with schizophrenia experienced constipation, indicating a potentially high prevalence compared with the general population.^1^, ^2^ It was higher than the constipation prevalence in schizophrenia patients, as reported by De Hart et al.^7^ It should be noted that this study may have underestimated the prevalence of constipation among schizophrenic patients, as it relied on self‐reported data. Patients with schizophrenia may have trouble reporting constipation on their own. Koizumi et al. discovered that only 18.5% of 184 inpatients with schizophrenia who met the diagnostic criteria for chronic constipation had reported it to their physicians out of a total of 533 inpatients.^16^ Patients with schizophrenia are unable to describe their pain sensation and lack awareness of their symptoms, causing subjective reports and scale ratings to underreport their symptoms; therefore, objective tests may be effective in identifying cases of gastroprokinetic hypomotility caused by antipsychotic‐induced constipation.^10^ It is possible that schizophrenic patients may have a higher incidence of constipation than what was found in this study if objective rating scales were used.

The multivariate analysis revealed a significant association between the female sex and constipation. Women with or without schizophrenia may be more susceptible to chronic constipation than men. According to a large cohort study conducted in the United States, women are more likely to develop chronic gastrointestinal disorders, including constipation, than men.^17^ Meta‐analyses have shown that chronic constipation is almost twice as common in women than in men.^2^ Various factors can cause constipation in women, such as hormonal changes during menstruation, increased risk of pelvic floor muscle dysfunction with age, decreased physical activity, comorbidities, prescription medications, dietary changes, and mental health concerns.^18^, ^19^, ^20^, ^21^ According to Horii et al., the difference in the incidence of constipation according to sex may be influenced by neurotransmitters supplied through the descending pain suppression pathway that differs based on sex.^22^

After conducting the multivariate analysis, we found a significant association between constipation and CDRs of antiparkinsonian drugs. Except for one case of amantadine, all of the antiparkinsonian drugs used by the patients in this study relied on anticholinergic effects. Anticholinergics are medications prescribed to treat extrapyramidal symptoms and prevent extrapyramidal side effects caused by antipsychotic medications. However, they can cause various peripheral side effects such as dry mouth, dysuria, and constipation, and central side effects such as cognitive impairment, worsening tardive dyskinesia, and delirium.^23^ Previous studies have shown that patients with a high anticholinergic burden have a high prevalence of constipation, indicating a potential link between anticholinergic load and constipation.^24^, ^25^, ^26^, ^27^, ^28^, ^29^ In this study, most antiparkinsonian drugs used exerted anticholinergic effects, which reduced extrapyramidal side effects. This study found that the CDRs of antiparkinsonian drugs were associated with constipation, indicating that antiparkinsonian drugs may affect constipation in a dose‐dependent manner.

The dose of BZD sleeping pills was significantly associated with constipation, as indicated by the CDR. BZD drugs with anticholinergic effects may increase the risk of constipation.^30^ A systematic review by Shinhuku et al. on the benefits of long‐term BZD treatment for anxiety disorders, eight randomized controlled trials (*n* = 1228) reported that BZDs were associated with higher incidence of constipation and dry mouth than placebos.^31^ Consideration should also be given to the possibility that BZDs may cause hypersedation and decreased locomotion. Virtanen et al. examined the factors associated with constipation in 275 patients with schizophrenia spectrum disorder who presented to an outpatient clinic.^32^ In the multivariate analysis, age, schizophrenia, institutionalization, and decreased regular physical activity were associated with constipation, except for psychotropic medication.^32^ Although not intended for patients with schizophrenia, in a cross‐sectional study conducted by Fosnes et al. on older patients living in nursing homes, constipation was found to be significantly associated with decreased activities of daily living, antidepressants (except for selective serotonin reuptake inhibitors), and BZD derivatives.^33^ Future studies should examine whether psychotropic medications indirectly exacerbate constipation by reducing physical activity.

Previous studies have suggested that antipsychotic medications may increase the risk of constipation.^34^, ^35^ However, the multivariate analysis in this study did not find any evidence to support this effect. Moreover, CDRs per drug showed no significant associations. Because this study only included patients with schizophrenia who were mostly taking antipsychotic medications, it was challenging to obtain statistically significant differences based on the presence or absence of antipsychotic medications. Another reason for the lack of significant differences could be that antiparkinsonian medications are more strongly associated with constipation than antipsychotics. However, it is possible that the number of cases was too small to draw any conclusions. Additionally, the strength of anticholinergic effects may vary among different antipsychotic medications in both SGAs and FGAs.^36^ Therefore, if antipsychotic medications are categorized as SGA or FGA, this association may be offset, resulting in a statistically significant decrease.

Nielsen et al. conducted a multivariate analysis of associated factors in 126 patients with ileus among 26 720 patients with schizophrenia. They found that factors significantly associated with ileus included older age, female sex, clozapine use, high‐dose FGA use, tricyclic antidepressant use, anticholinergic drug use, and opioid use.^37^ Given the high rate of constipation in patients with schizophrenia in this study (43.4%), the possibility that antipsychotic medications are among the factors that increase the risk of constipation in patients with schizophrenia should be considered when administering drug therapy.

One possible explanation for the high prevalence of constipation in individuals with schizophrenia in this study is that lifestyle factors may be associated with schizophrenia. Dipasquale et al. reported that individuals with schizophrenia tend to have an unhealthy diet, high in saturated fat and low in fiber and fruit.^38^ A study by Tucker et al. showed that increased fiber intake results in large stools and quick passage of waste through the colon, whereas a low‐fiber diet can cause constipation.^39^ In addition, people with severe mental illness (e.g., schizophrenia, schizoaffective disorder, other psychotic disorders, bipolar disorder, or major depressive disorder) engage in less vigorous exercise and more sedentary behaviors than healthy controls.^40^, ^41^, ^42^ Inadequate fiber and fluid intake, as well as reduced physical activity, are known risk factors for constipation in patients with schizophrenia.^43^ Several reports have suggested that inactivity increases the risk of constipation in the general population.^44^, ^45^, ^46^, ^47^ Changes in diet and exercise habits associated with schizophrenia may increase the overall risk of constipation. Future studies should examine lifestyle factors in individuals with schizophrenia to understand their impact on constipation.

The study found no link between exercise habits and constipation. This could be because the study used a cross‐sectional self‐administered questionnaire, which didn't provide enough detail about the quality and intensity of exercise. In the future, a prospective study that equalizes the intensity and duration of exercise will be needed to understand the effects of exercise on constipation in schizophrenic patients more clearly. Additionally, the study did not find a significant association between smoking and constipation. Further examination of the effect of smoking on constipation is needed. This is because smoking may alleviate constipation due to its parasympathomimetic effects^48^ and by reducing blood levels of other psychotropic drugs through the induction of liver enzymes.^49^

The CDRs used in this study are valuable indicators of dosage. Its advantages include the ease of calculation and applicability to any drug with an upper limit specified on the package insert. In addition, the OR in the multivariate analysis represented the relationship when the upper limit was used for approximately one drug, making it simple to visualize the dosage. The ORs for analyzing multiple variable doses can be helpful for medicines that do not have indices, such as chlorpromazine equivalents for antipsychotic medications, imipramine equivalents for antidepressants, and diazepam equivalents for BZD drugs. However, it is crucial to note that CDRs are not indices based on pharmacological effects and deviate from the above conversion values. Drug action characteristics, metabolic pathways, and how individual constituents metabolize them are not always identical. Therefore, the significance of CDRs should only be considered as a reference method in this study to examine the dose‐dependent associations. In the future, we aim to assess the effectiveness of the CDR, including its alignment with other conversions.

It is crucial to acknowledge that constipation is a severe medical condition that has adverse effects on one's quality of life. Furthermore, it may also be a significant contributor to the unfavorable prognosis of individuals with schizophrenia. It is essential to conduct high‐quality research in the future to examine the correlations between schizophrenia, drug therapy, and constipation. Individuals with schizophrenia are also likely to have physical illnesses that affect their health.^50^ In the treatment of schizophrenia, it is crucial to raise awareness of physical complications, such as constipation, and to aim for the early prevention and detection of these issues.

This study has some limitations. First, this was a cross‐sectional study that could not determine cause‐and‐effect relationships. Second, although we examined many variables, we did not investigate all the lifestyle factors, including meal details, exercise quality, and water intake. Third, since the questionnaires were self‐reported, the findings may not accurately reflect true lifestyles and habits. Fourth, the CDR used in this study was a unique indicator. Fifth, this study did not examine the duration of internal drug use. Finally, this study was unable to examine the impact of physical complications, including the history of abdominal surgery. These limitations may have restricted our discussion.

## 5 CONCLUSIONS

The findings of this study indicate that constipation is common in outpatients with schizophrenia. Furthermore, the study suggests that female gender and use of antiparkinsonian medications and BZD sleep medications, which act in a dose‐dependent manner, may be associated with constipation.

## AUTHOR CONTRIBUTIONS

Taro Tazaki designed the study, wrote the protocol, collected the data, performed the statistical analyses, and wrote the manuscript. Hiroki Yamada and Osamu Takashio wrote the protocol, collected the data, performed the statistical analysis, and verified the manuscript. Ryotaro Sato, Hiroki Ishii, Shutarou Sugita, Haruka Yanagihara, and Dan Nakamura collected data and confirmed the manuscript. Atsuko Inamoto and Akira Iwanami critically revised the manuscript for intellectual content. All authors contributed to the manuscript and approved this submission.

## FUNDING INFORMATION

The authors received no grants.

## CONFLICT OF INTEREST STATEMENT

TT declares no conflict of interest. HY has received advisory panel payments from Yoshitomi Yakuhin and speaker's honoraria from Astellas Pharmaceutical, Eisai, Janssen Pharmaceuticals, Lundbeck Japan, Meiji‐Seika Pharma, Mochida Pharmaceutical, Otsuka Pharmaceutical, Sumitomo Pharma, Viatris Pharmaceutical, and Yoshitomi Yakuhin within the past 3 years. RS, HI, SS, and HY declare no conflict of interest. DN has received speaker's honoraria from Janssen Pharmaceuticals, Meiji‐Seika Pharma, Otsuka Pharmaceutical, Sumitomo Pharma, Takeda Pharmaceutical, and Viatris Pharmaceutical within the past 3 years. OT has received speaker's honoraria from Meiji‐Seika Pharma, Otsuka Pharmaceutical, Sumitomo Pharma, Viatris Pharmaceutical, and Takeda Pharmaceutical within the past 3 years. AI [Atsuko Inamoto] has received a research grant from MSD and speaker's honoraria from Daiichi Sankyo, Eisai, MSD, Otsuka Pharmaceutical, Sumitomo Pharma, Viatris Pharmaceutical, and Yoshitomi Yakuhin within the past 3 years. AI [Akira Iwanami] received research grants from Daiichi Sankyo, Eisai, Mochida Pharmaceutical, Otsuka Pharmaceutical, Shionogi Pharmaceutical, Sumitomo Pharma, and Takeda Pharmaceutical within the past 3 years.

## ETHICS STATEMENT

Approval of the research protocol by an Institutional Reviewer Board: The protocol for this research project has been approved by the Ethics Committee on Research Involving Human Subjects at Showa University (22‐067‐B). The protocols were performed according to the “Ethical Guidelines for Medical Research Involving Human Subjects” established by the Ministry of Health, Labour and Welfare and the Ministry of Education, Culture, Sports, Science and Technology in Japan.

Informed consent: Written informed consent was obtained from all participants before administering the questionnaire, and information was extracted from their medical records. A clear explanation regarding the writing of the study and the fact that the participants could refuse to participate at any point was provided to each participant and notified on our website. Personal information was anonymized using numerical codes, and a designated information manager was appointed to oversee its management. The list of individuals and their corresponding anonymized numbers was stored on an in‐hospital server separated from the external network, and access was restricted using password settings.

Registry and the Registration No. of the study/trial: N/A.

Animal Studies: N/A.

Permission to reproduce material from other sources: N/A.

## Data Availability

Raw data were generated at the Department of Psychiatry at Showa University. Derived data supporting the findings of this study cannot be disclosed, for which patients' agreement was not acquired through informed consent. Part of it will be available from the corresponding author upon request.
